# Editorial: Neural Interface for Cognitive Human-Robot Interaction and Collaboration

**DOI:** 10.3389/fnins.2022.830877

**Published:** 2022-03-03

**Authors:** Rui Huang, Lian Zeng, Hong Cheng, Xiaodong Guo

**Affiliations:** ^1^School of Automation Engineering, University of Electronic Science and Technology of China, Chengdu, China; ^2^Department of Orthopedics, Union Hospital, Tongji Medical College, Huazhong University of Science and Technology, Wuhan, China

**Keywords:** BCI, brain-computer interface, human-robot collaboration, EEG, exoskeleton, action recognition, rehabilitation, human-robot interaction

Human-robot interaction (HRI) and collaboration is a major topic in human-coupled robotic systems. With the development of neural technologies, the neural interface called cognitive human-robot interaction has been implemented for achieving natural human-robot interaction and collaboration. This special issue will be dedicated to cognitive human-robot interactions, including brain-computer interface (BCI) with electroencephalographic (EEG), muscle signals with electromyography (EMG), and so on. The special issue is focused on the fundamentals and technologies of cognitive human-robot interaction with neural interfaces for human-coupled robotic systems. Ten articles were accepted by this special issue, the contents of these articles are briefly described as follows.

As the development of electroencephalographic techniques for commercial applications continues, their transformative potential necessitates equally significant ethical inquiries. Lopez et al. consult different databases, which presents conceptual and empirical discussions and findings about various commercial and ethical aspects of electroencephalography. Subsequently, the content is extracted from the articles and the main conclusions are presented. Finally, an external assessment of the outcomes is conducted in consultation with an expert panel in some of the topic areas such as biomedical engineering, biomechatronics, and neuroscience.

Sensorimotor rhythm (SMR)-based BCIs can help users perform motor control using motor imagery. But the control paradigm of SMR BCI may not work well on a subpopulation of users. Jiang et al. investigate the behavioral and electrophysiological differences between experienced meditators and meditation naïve subjects in one-dimensional and two-dimensional cursor control tasks. The evidence shows that meditators outperformed control subjects in both tasks. Further, the meditators had a higher resting SMR predictor, more stable resting mu rhythm, and a larger control signal contrast than controls during the task.

In dynamic manufacturing and warehousing environments, workers suffer from muscle fatigue of the lower limbs caused by standing or squatting for a long period of time. Wang Z et al. design and evaluate a semi-active lower-limb exoskeleton to reduce muscle fatigue. The exoskeleton can switch three different modes depending on the EMGs of the gluteus maximus, and quadriceps. Three sets of experiments are conducted to evaluate the effect of the exoskeleton, results show that the exoskeleton not only effectively reduced muscle fatigue but also avoided interfering with the free movement of the wearer.

Exoskeleton rehabilitation robots can help paraplegics motor functions. However, achieving stable states of the human-exoskeleton system while conserving wearer strength remains challenging. Wang C et al. propose a real-time stable control gait switching method for the exoskeleton rehabilitation robot. The method uses surface electromyography (sEMG)-based HRI to realize the intention recognition, and realizes gaits planning and stability analysis based on a human kinematics model and zero moment point method. Results verified the feasibility and efficiency of the proposed gait switching method for enhancing stability and ergonomic effects of a lower limb rehabilitation exoskeleton.

The features extracted from EEG usually change dramatically, but emotion states change gradually. Most existing feature extraction approaches do not consider these differences between EEG and emotion. Chen et al. propose a novel feature extraction method based on EEG microstates for emotion recognition. This method extracts microstate characteristics as novel temporospatial features, and the dual-threshold-based atomize and agglomerate hierarchical clustering method is used to determine the optimal number of microstate classes automatically. Results indicated that microstate characteristics can effectively improve the performance of emotion recognition from EEG signals.

The root mean square (RMS) of the sEMG signal displays a positive correlation with muscle force and muscle tension under positive and passive conditions, respectively. Li et al. investigate the changes in muscle force and tension after multilevel surgical treatments, functional selective posterior rhizotomy, and tibial anterior muscle transfer surgery, and evaluate their clinical effect in children with spastic cerebral palsy (SCP) during walking. Results indicate that the neuromuscular function of cerebral palsy during walking can be evaluated by the muscle activation state and the RMS of the sEMG signal, which shows that multilevel surgical treatments are feasible and effective to treat SCP.

In the field of motor imagery (MI) classification, appropriate filtering is vital for feature extracting of EEG signals and consequently influences the accuracy of MI classification. Yan et al. propose a novel two-stage refine filtering method, it uses the gradients of any target concept flowing into the final convolutional layer to highlight the important part of training data for predicting the concept. Experiment results reveal that the proposed method reaches state-of-the-art classification kappa value levels and acquires at least 3% higher kappa values than other methods.

The generalization goals of most skill expression models in real scenarios are specified by humans or associated with other perceptual data. Guan et al. propose a framework using the Probabilistic Movement Primitives modeling to improve the robot stiffness-adaptive skill primitive generalization. It uses sEMG signal to estimate human arm endpoint stiffness, which can then be transferred to the robot. The proposed framework can be used to trigger robot action generalization *via* observing human action, ideal for a human-robot collaboration scenario.

Improving human motor performance *via* physical guidance by an assist robot device is a major field of interest for society in many different contexts, such as rehabilitation and sports training. Takai et al. propose a Bayesian estimation method to predict whether the motor performance of a user can be improved from the initial skill level of a user or not *via* robot guidance. Results show that the proposed approach can potentially help users to decide if they should try a robot-guided training or not without conducting the time-consuming robot-guided movement training.

The HRIs have been widely used in exoskeleton robots to help predict the movement of the wearer, especially sEMG-based HRIs. But the sEMG signals from paraplegic patients' lower limbs are weak, which means an HRI based on lower limb sEMG signals cannot be applied to exoskeletons. Shi et al. propose an HRI based on upper limb sEMG signals to predict the lower limb movements of paraplegic patients. The interface constructs a channel synergy-based network to extract the contribution and synergy of different feature channels. Results show that the method has a good movement prediction performance in both within-subject and cross-subject situations.

## Author Contributions

RH and LZ drafted the manuscript. XG and HC critically revised the manuscript. All authors approved the submitted version.

## Funding

This work was supported by the National Natural Science Foundation of China (grant nos. 82072446 and 81873999) and the Key R&D Program of Hubei Province (2020BCB050).

## Conflict of Interest

The authors declare that the research was conducted in the absence of any commercial or financial relationships that could be construed as a potential conflict of interest.

## Publisher's Note

All claims expressed in this article are solely those of the authors and do not necessarily represent those of their affiliated organizations, or those of the publisher, the editors and the reviewers. Any product that may be evaluated in this article, or claim that may be made by its manufacturer, is not guaranteed or endorsed by the publisher.

